# A new model to estimate duration of survival in patients with hepatocellular carcinoma with BCLC intermediate stage

**DOI:** 10.1038/s41598-023-48068-7

**Published:** 2023-11-25

**Authors:** Masashi Ninomiya, Mio Tsuruoka, Jun Inoue, Atsushi Hiraoka, Tomoaki Iwata, Akitoshi Sano, Kosuke Sato, Masazumi Onuki, Satoko Sawahashi, Hidekatsu Kuroda, Takayoshi Oikawa, Masashi Fujita, Kazumichi Abe, Tomohiro Katsumi, Wataru Sato, Go Igarashi, Chikara Iino, Tetsu Endo, Nobukazu Tanabe, Hiroshi Numao, Katsunori Iijima, Takayuki Matsumoto, Hiromasa Ohira, Yoshiyuki Ueno, Atsushi Masamune

**Affiliations:** 1 Division of Gastroenterology, Tohoku University Graduate School of Medicine, 1-1 Seiryomachi, Aobaku, Sendai, Miyagi 9808574 Japan; 2 Gastroenterology Center, Ehime Prefectural Central Hospital, Matsuyama, Ehime Japan; 3 Division of Gastroenterology and Hepatology, Department of Internal Medicine, Iwate Medical University, Shiwa, Iwate Japan; 4 Department of Gastroenterology, Fukushima Medical University School of Medicine, Fukushima, Japan; 5 Department of Gastroenterology, Yamagata University Faculty of Medicine, Yamagata, Japan; 6 Department of Gastroenterology, Graduate School of Medicine, Akita University, Akita, Japan; 7 Department of Gastroenterology, Hirosaki University Graduate School of Medicine, Hirosaki, Japan; 8 Department of Gastroenterology, National Hospital Organization Sendai Medical Center, Sendai, Japan; 9 Department of Gastroenterology, Aomori Prefectural Central Hospital, Aomori, Japan

**Keywords:** Liver cancer, Cancer models

## Abstract

It is difficult to determine whether an individual therapy contributes to the elongation of survival because of the difficulty of organizing clinical research in patients who receive multiple treatments in HCC. We aimed to establish a new model of survival prediction in patients with intermediate stage HCC to establish standards in the recent and coming multi-MTA era. This analysis was prepared using a data set of 753 patients diagnosed HCC prior to 2017. Multiple regression analysis showed age, naïve or recurrence, the size of the largest tumor nodule, the number of nodules, total bilirubin, albumin and α-fetoprotein as independent predictors of survival. A Weibull model had the best fit and, based on these predictors, we established a new predicted survival model. The survival duration can be predicted the proposed model; EXP (4.02580 + (− 0.0086253) × age + (− 0.34667) × (naïve/recurrence) + (− 0.034962) × (number of nodules) + (− 0.079447) × (the size of the largest nodule) + (− 0.21696) × (total bilirubin) + 0.27912 × (albumin) + (− 0.00014741) × (α-fetoprotein)) × (− natural logarithm(0.5))^0.67250. This model is useful for the planning and evaluating the efficacy of recent sequential therapies in multi-MTA era.

## Introduction

Hepatocellular carcinoma (HCC) is the most common primary liver neoplasm and the second leading cause of cancer-related mortality worldwide^1^. HCC is now generally treated according to the modified Barcelona Clinic Liver Cancer (BCLC) staging system and the treatment strategy recommended by European Association for the Study of the Liver (EASL) guidelines. BCLC staging system are classified into five stages and intermediate stage categorized by medium position. It is defined as multinodular by tumor status, preserved liver function and 0 for performance status^1^. Transcatheter arterial chemoembolization (TACE) is recommended as the first-line treatment and median survival was slightly overed 20 months by randomized controlled trial^2^. Later some previous cohort studies have reported a median survival of around 40 months in well selected candidates with a good technique approach^3,4^. In 2008, sorafenib was the first tyrosine kinase inhibitor (TKI) approved for use in patients with unresectable HCC. The group of patients who received sorafenib survived about 3 months longer than those who did not receive the drug^5^. A trial that compared the efficacy of TACE plus sorafenib with TACE alone was conducted in Japan and it was reported that TACE plus sorafenib significantly improved progression free survival (PFS) compared to TACE alone in patients with intermediate stage HCC^6^. After 2017, some molecular-targeted agents (MTAs) and immunotherapy became available in Japan^7–11^. Patients with intermediate stage HCC represent various types with regard to tumor burden and liver function status^12^. Some populations in this stage have been included among cases with TACE failure or refractoriness. Therefore, sequential therapy of some MTAs or combination therapy with TACE and MTAs have been proposed in subpopulations of intermediate stage HCC^13,14^. Individual MTA therapy has been reported to show efficacy, but it is not easy to achieve a complete response. Even if a partial response can be achieved, tumor progression and/or new lesions may sometimes occur due to resistance to the previous therapy. Thus, sequential MTA treatment or MTA-TACE therapy has been administered and reported to prolong survival^6,15–18^. Nowadays, multi-MTA has become the usual HCC therapy, whereas Sorafenib was the predominant therapy in TACE refractory patients before 2017. But there are still some problems. One of the problems is that it is difficult to evaluate the appropriateness of the sequential systemic therapy for each patient, because of the difficulty in organizing highly evidenced clinical studies for too many sequential and/or combined treatments.

If we can predict the survival of one HCC patient treated with the previous therapy, especially prior to 2017, which included only TACE or sorafenib, treating a patient with the current sequential MTA treatment and and/or MTA-TACE therapy will enable us to evaluate the effectiveness. So far, several factors related to the prediction of HCC patients’ survival have been reported, such as gender, age, tumor morphology, the grade of liver function and the value of tumor markers^19–22^. The prediction of HCC patients’ survival is important for therapeutic management. Parametric models are generally used in survival studies for accurate predictions. Some of the parametric models have suggested an acceleration of the failure time approach, which directly targets the patients’ survival prediction^23^. Therefore, using parametric models could provide a better estimate to predict the duration of survival.

In this study, we investigated the predictors of survival in the patients with intermediate stage HCC before 2017 and developed a mathematical model to estimate the survival using a parametric distribution.

## Methods

### Study design and participants

In this retrospective cohort study, 753 HCC patients were included 2002 to 2017 at the eight liver centers in Japan (Akita University Hospital, Iwate Medical University Hospital, Tohoku University Hospital, Hirosaki University Hospital, Aomori Prefectural Central Hospital, Yamagata University Hospital, Fukushima Medical University Hospital and Ehime Prefectural Central Hospital). HCC stage was evaluated according to BCLC classification and all the cases when diagnosed as intermediate stage was enrolled in this cohort. The clinical information of patients was extracted from their medical records and they were followed to identify their death status via phone-call or medical information sheet from their relative hospitals. The survival time was designated as the time between the diagnosis date of HCC intermediate stage and the occurrence of death. The death status was considered as a failure event. All therapies were allowed but just one type of MTA (sorafenib) could be used. No patients underwent liver transplantation.

This study was approved by the institutional review board of Tohoku University Hospital (2021-1-377). The study was conducted in accordance with the principles of the Declaration of Helsinki (Fortaleza revision, 2013).

### Data collection

The HCC nodules were characterized by contrast-enhanced computed tomography (CT) or magnetic resonance imaging (MRI), including the number of tumor nodules, the diameter of the largest nodule and the vascular invasion. The following clinical parameters and biochemistry data were included in the table: age, gender, etiology, Eastern Cooperative Oncology Group (ECOG) performance status, total bilirubin, aspartate aminotransferase (AST), alanine aminotransferase (ALT), albumin, platelets, prothrombin time, α -fetoprotein (AFP), des-γ-carboxyprothrombin (DCP), tumor size and numbers, Child–Pugh score, albumin-bilirubin (ALBI) score and modified ALBI (mALBI) grade, Kinki criteria, tumor node metastasis-the liver cancer study group of Japan (TNM-LCSGJ) and treatment naïve or recurrence. The ALBI score was calculated using the formula: linear predictor = (log_10_ (total bilirubin × 17.1) × 0.66) + (albumin × 10 × − 0.085), and the cut points of the mALBI grade were as follows: x ≤ − 2.60 (grade 1), more than − 2.60 to < − 2.27 (grade 2a), not less than − 2.27 to ≤ − 1.39 (grade 2b) and more than − 1.39 (grade 3)^24,25^. Kinki criteria was subclassified into three stages in the BCLC intermediate stage. It was classified as follows: Child–Pugh scores of 5–7 points with ‘in’ in terms of the ‘up-to-seven’ criteria (B1), Child–Pugh scores of 5–7 points with ‘out’ of the ‘up-to-seven’ criteria (B2) and Child–Pugh scores of 8–9 points with either ‘in’ or ‘out’ of the ‘up-to-seven’ criteria (B3)^26,27^. Continuous variables are presented as the mean ± standard deviation or median (interquartile range) and categorical variables as numbers. Survival was calculated as the time from the date of the initial diagnosis as BCLC intermediate stage to death by HCC.

### Statistical analysis and development of parametric models

Patient survival probability was analyzed using the Kaplan–Meier method. The survival-related factors were extracted by the Cox proportional-hazard regression model. Based on the factors with multivariate significance (*p* < 0.05) and clinical relevance that have been previously reported.

Parametric models were applied and we selected three parametric models including the exponential, Weibull and log-normal. The fit of the models was evaluated using probability plots and the Akaike information criterion (AIC) and Bayesian information criterion (BIC), with a smaller value indicating a better fit. The AIC and BIC are methods based on in-sample fit to estimate the likelihood of a model to calculate the future values^28,29^. A plot of the negative log of the estimated survivor function against log time can provide a visual check of the appropriateness of the parametric model for the survival data^30^.

### Weibull distribution model

The survival model was applied based on Weibull distribution. The cumulative failure probability was defined as F (x, α, β) = 1 − exp[− (x/β)^α^]. x showed the survival time, α, scale parameter and β, shape parameter. In this case, median survival time could be calculated as follows

$$ \begin{aligned} & {\text{F }}\left( {{\text{x}},\alpha,\beta} \right) \, = { 1} - {\text{ exp}}\left[ { - \left( {{\text{x}}/\beta} \right)^{\alpha} } \right] \, = \, 0.{5} \\ & {\text{x }} = \beta{\text{x }}\left( { - {\text{log}}0.{5}} \right)^{{{1}/\alpha}} \\ \end{aligned} $$

β is consisted of the statistical weighing and the risk factors.

$$ {\beta } = {\text{ exp }}\left( {{\text{intercept }} + {\text{ coefficient1}}*\left( {{\text{factor1}}} \right) \, + {\text{ coefficient2}}*\left( {{\text{factor2}}} \right) + \cdots } \right) $$

The risk factors were extracted from the multivariate analysis of the Cox proportional-hazard regression model. The intercept and the coefficient of each risk factor were determined by JMP Pro 15.0.0 (SAS Institute, Cary, NA).

### Ethical approval

This study followed the principles of the Declaration of Helsinki (Fortaleza revision, 2013). Study approval statement: This study was reviewed and approved by the institutional review board of Tohoku University Hospital (approval number: 2021-1-377).

### Consent to participate

Due to the retrospective observational study, the institutional review board of Tohoku University Hospital waived the need for written informed consent. The identifying data of the enrolled patients has been delinked and the authors could not access the individual data.

## Results

### The characteristics of the patients in this cohort study

**Figure 1:**
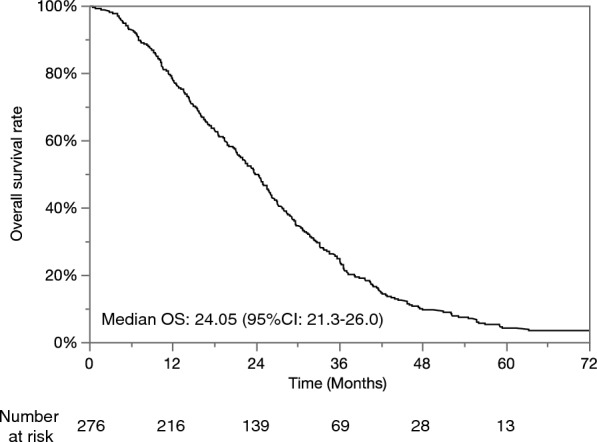
Kaplan–Meier curves for overall survival in this cohort study.

The characteristics of the patients in the cohort are shown in Table 1. 753 patients were enrolled in this cohort. The median age was 70 years and the majority were male. Hepatitis C virus was the predominant etiology. All patients had normal performance status. There were 300 (39.8%) patients with a Child–Pugh score of 5, 242 (32.1%) with a score of 6, 118 (15.7%) with a score of 7 and 93 (12.4%) with a score of over 8, corresponding to 542 (72.0%) patients with Child–Pugh class A and 211 (38.0%) with up to class B. The median ALBI score was − 2.22, There were 184 (24.4%) patients with mALBI grade 1, 150 (19.9%) with grade 2a, 355 (47.1%) with grade 2b, and 64 (8.5%) with grade 3. The median size of the largest nodule was 3.0 cm and the number of nodules was five. According to Kinki criteria, there were 279 (37.1%) patients with B1 stage, 381 (50.6%) with B2 and 93 (12.3%) with B3. The median AFP concentration was 39.6 ng/dL and DCP was 111 mAU/mL. When diagnosed HCC with intermediate stage, 279 (37.1%) patients were treatment naïve, while 474 (62.9%) were recurrence. After the BCLC intermediate stage, the median overall survival (OS) was 24.05 months by the non-parametric estimator of survival functions (Fig. 1).

**Table 1:** Description of the patients (n = 753).

Patient characteristics	Value	Tumor characteristics	Value
Age, years	70 (63–76)	Kinki criteria	
Gender		B1	279
Male	553	B2	381
Female	200	B3	93
Etiology		TNM-LCSGJ	
HCV	516	II	205
HBV	71	III	548
HCV + HBV	15	Size of the largest nodules, cm	3 (1.9–4)
nonBnonC	151	< 1	2
ECOG, PS 0/1	753/0	1–2	190
Total bilirubin, mg/dL	0.9 (0.6–1.3)	2–3	168
AST, U/L	54 (37–75)	3–4	179
ALT, U/L	41 (26.75–63)	4–6	135
Albumin, g/dL	3.5 (3.1–3.9)	6–10	63
Platelets, × 10^4^/μL	10.1 (7.1–13.9)	≥ 10	16
Prothrombin time, %	85 (73.5–97.9)	Number of nodules	5 (3–7)
ALBI score	-2.22 (-2.59—-1.79)	2	127
mALBI grade		3	81
1	184	4	167
2a	150	5	95
2b	355	6–7	100
3	64	8–9	35
Child–Pugh score		≥ 10 or diffuse	148
5	300	Naïve/recurrence	279/474
6	242	AFP, ng/dL	39.6 (10.2–262.5)
7	118	DCP, mAU/mL	111 (29–728.75)
≥ 8	93		

*HCV* hepatitis C virus, *HBV* hepatitis B virus, *ECOG* Eastern Cooperative Oncology Group, *PS* performance status, *AST* aspartate aminotransferase, *ALT* alanine aminotransferase, *ALBI* albumin-bilirubin, *mALBI* modified ALBI, *TAE* transcatheter arterial embolization, *TACE* transcatheter arterial chemoembolization, *TKI* tyrosine kinase inhibitor, *TNM-LCSGJ* tumor node metastasis-the liver cancer study group of Japan, *AFP* α-fetoprotein, *DCP* des-γ-carboxyprothrombin.

### Selection of parametric models

**Figure 2:**
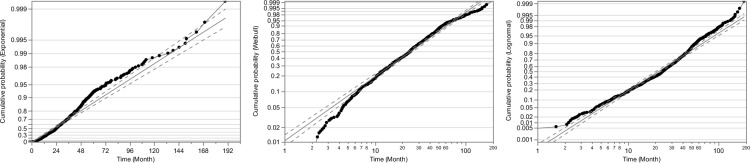
Estimation of parametric model. Probability plots for each distribution. The linear course indicates the graph of log–log survival against the log of failure time in each distribution. The dot-to-dot linear showed the survival distribution.

We compared the performance in which we fit each model. The fit of the models was compared using AIC and BIC, and the Weibull distribution showed a smaller value (Table 2). The probability plots were prepared regardless of whether or not the data set followed a given distribution such as the three parametric models. The probability plots can provide a visual check of the appropriateness. The Weibull plots appeared approximately linear (Fig. 2).

**Table 2:** The score of Akaike information criterion and Bayesian information criterion.

Distribution	AIC	BIC
Exponential	6597.3596	6601.9783
Weibull	6515.0951	6524.3272
Lognormal	6535.6939	6544.9261

*AIC* Akaike information criterion, *BIC* Bayesian information criterion.

### Predictor of survival and survival prediction model.

A univariate Cox proportional-hazard regression analysis was performed in this cohort (Table 3). Age, naïve or recurrence, sum of the size of the largest tumor nodule, the number of nodules, total bilirubin, albumin, AFP and DCP were significantly different. Based on these variables, multiple regression analysis was conducted. Age, naïve or recurrence, sum of the size of the largest tumor nodule, the number of nodules, total bilirubin, albumin, AFP and DCP were selected as independent predictors of survival (Table 3).

**Table 3:** Univariate and multivariate analyses of derivation cohort by the Cox proportional-hazard regression model.

Factors	Category	Univariate		Multivariate	
		Hazard ratio	*P* value	Hazard ratio	*P* value
Age	Years	2.449 (1.419–4.089)	0.0016	2.132 (1.164–3.939)	0.0140
Gender	Male/ Female	0.836 (0.710–0.983) 1.197 (1.018–1.408)	0.0324		
Etiology	Viral/ Nonviral	0.835 (0.698–1.000) 1.198 (1.000–1.433)	0.0531		
Treatment	Naïve/ Recurrence	0.729 (0.627–0.848) 1.371 (1.179–1.595)	< 0.0001	1.450 (1.252–1.801)	< 0.0001
Number of nodules	Variable	1.471 (1.186–1.818)	0.0005	1.670 (1.305–2.128)	< 0.0001
Size of the largest nodule	Variable	2.582 (1.529–4.269)	0.0005	4.100 (2.319–7.135)	< 0.0001
Total bilirubin	Variable	5.388 (2.925–9.639)	< 0.0001	5.304 (2.615–10.50)	< 0.0001
Albumin	Variable	0.294 (0.201–0.431)	< 0.0001	0.307 (0.194–0.485)	< 0.0001
Platelets	Variable	0.475 (0.150–1.359)	0.1720		
Prothrombin time	Variable	1.066 (0.624–1.813)	0.8152		
AFP	Variable	191.6 (42.91–629.2)	< 0.0001	66.45 (11.86–246.8)	< 0.0001
DCP	Variable	7.152 (3.017–15.34)	< 0.0001	3.779 (1.463–9.000)	0.0038

*P* value < 0.05 denotes statistical significance.

*AFP* α-fetoprotein, *DCP* des-γ-carboxyprothrombin.

### Development of a new estimated survival model

We developed a new mathematical survival model using Weibull distribution with seven predictors selected by multiple regression analysis. In this cohort, DCP was included the independent predictor. But we could not measure the DCP when patient indicated warfarin, and therefore we determined not to recommend as the risk factor. There were still two issues in making this model. One was how to count the nodules of the numerous or diffuse type accurately. Up to ten nodules, we usually could not count them accurately. For this, one of the criteria with liver transplantation showed that fewer than 11 nodules could indicate transplantation. Therefore, we set eleven when the number of HCC nodules were more than 10 or the diffuse type. Next was how to manage the value of AFP when it was too wide to use a parameter. The outlier could hardly be indicated as a parameter for the mathematical model. Therefore, we designated the outlier as outside the 95% confidential interval (CI). Greater than 5078 ng/dL was out of 95%CI and we set 5078 as the value of AFP when AFP was over 5078. In Weibull distribution model, the predicted survival time was derived as [exp (intercept + coefficient1 × (factor1) + coefficient2 × (factor2) + …) × (− log 0.5)^1/α^]. The seven factors related with overall survival were selected by Cox proportional-hazard regression analysis. The parameter estimates were calculated by JMP program and shown in Table 4. The 50% survival duration (months) was predicted by this new Weibull model indicated as EXP(4.02580 + (− 0.0086253) × Age + (− 0.34667) × (0 for naïve/1 for recurrence) + (− 0.034962) × (number of nodules) + (− 0.079447) × (the size of the largest nodule, cm) + (− 0.21696) × (T-bil, mg/dl) + 0.27912 × (Alb, g/dl) + (− 0.00014741) × (AFP, ng/dl)) × (− log0.5)^0.67250 (supple info. 1).

**Table 4:** The coefficient of each factor in Weibull survival model.

Factors	Input variable	Coefficients	Standard error
Intercept		4.02580	0.30824
Age	Years	− 0.0086253	0.0030105
Naïve/recurrence	Naïve: 0/Recurrence: 1	− 0.34667	0.056452
Number of nodules	Under 10: numbers	− 0.034962	0.0084603
	Over 10: 11		
	Diffuse type: 11		
Size of the largest nodule	Size (cm)	− 0.079447	0.013491
Total bilirubin	Value (mg/dl)	− 0.21696	0.041451
Albumin	Value (g/dl)	0.27912	0.048091
AFP	Under 5078: each value	− 0.00014741	0.000019594
	Over 5078: 5078		
1/α		0.67250	0.018108

*AFP* α-fetoprotein.

## Discussion

In this study we demonstrated the predictors of survival in intermediate stage HCC patients and developed a mathematical model to estimate the survival time based on the predictors of the parametric distribution. The Weibull distribution had the best fit among all investigated parametric models in our data. The EASL guidelines recommend treating intermediate stage HCC patients with TACE and showed 2.5 years as the estimated mean survival time. After 2017, several MTAs and one immune checkpoint inhibitor combined with a single MTA were approved for HCC therapy in Japan. Each MTA was confirmed for its effectiveness based on the overall survival and/or progression free survival in several randomized, double-blind clinical trials. However, even treatment with atezolizumab and bevacizumab for unresectable HCC patients shows complete remission in only about 5% of patients. Therefore, in the real world, multiple treatments such as combination and/or sequential therapy with MTAs, TACE and radiation are usually administered for HCC patients in the intermediate stage. Actually, it is difficult to evaluate the effectiveness of sequential total systemic therapy. Given these circumstances, we developed a mathematical model to estimate the survival duration in intermediate stage HCC patients. Using this new model, we can evaluate the efficacy of recent sequential therapy to determine whether it is appropriate or not.

TACE was recommended only for intermediate stage HCC patients before 2018. Several studies have reported predictors or predicted models. The parameters of tumor burden, liver function reserves and AFP are well-known predictors of survival in intermediate stage HCC patients undergoing TACE^19,26,31–33^. The intermediate stage subclassification system adopted in the Child–Pugh score included up-to-seven criteria^31^. Recent studies suggest that the ALBI grade might be a better surrogate of liver function reserve in HCC patients treated with TACE^34^. Moreover, some prediction models that rely on post-TACE assessment have been reported, but they are not useful for the selection of treatment^33,35^. To develop a predicted survival model, we investigated the predictors of overall survival. By multivariate analysis, we identified naïve/recurrence, number of nodules, size of the largest tumor, total bilirubin, albumin and AFP levels as independent predictors of overall survival in intermediate stage HCC patients. Based on these predictors, our survival model was developed using these parameters. This model is consistent with all routinely available parameters and is simple to calculate by common calculation software.

We established the predictors of survival in HCC patients through a parametric survival modeling approach. Previously, parametric models were well known for analyzing survival data. Survival time is considered to follow known distributions in exponential, Weibull and lognormal models^23^. Some parametric models that identified survival predictors of some diseases have been described. A Weibull or lognormal distribution is typically used for survival predictors^36–39^. In our data, The Weibull model had the best fit among the three investigated parametric models for the AIC score. Interestingly, using our new parametric survival model, we achieved more flexibility for predicting the survival duration in patients with intermediate stage HCC. This model could be recommended for planning, health policy-making and the evaluation of treatments and, potentially, it may contribute to improving the survival of patients with HCC.

### Supplementary Information

Supplementary Information.

## Data Availability

All data generated or analysed during this study are included in this article. Further enquiries can be directed to the corresponding author.

## Abbreviations

AFP: α-Fetoprotein
AIC: Akaike information criterion
ALBI: Albumin-bilirubin
ALT: Alanine aminotransferase
AST: Aspartate aminotransferase
BCLC: Barcelona clinic liver cancer
BIC: Bayesian information criterion
CT: Contrast-enhanced computed tomography
DCP: Des-γ-carboxyprothrombin
EASL: European Association for the Study of the Liver
ECOG: Eastern Cooperative Oncology Group
HCC: Hepatocellular carcinoma
mALBI: Modified ALBI
MRI: Magnetic resonance imaging
MTAs: Molecular-targeted agents
PS: Performance status
TACE: Transcatheter arterial chemoembolization
TAE: Transcatheter arterial embolization
TKI: Tyrosine kinase inhibitor
TNM-LCSGJ: Tumor node metastasis-the liver cancer study group of Japan
